# PPARs: Nuclear Receptors Controlled by, and Controlling, Nutrient Handling through Nuclear and Cytosolic Signaling

**DOI:** 10.1155/2010/435689

**Published:** 2010-08-01

**Authors:** Maria Moreno, Assunta Lombardi, Elena Silvestri, Rosalba Senese, Federica Cioffi, Fernando Goglia, Antonia Lanni, Pieter de Lange

**Affiliations:** ^1^Dipartimento di Scienze Biologiche ed Ambientali, Università degli Studi del Sannio, Via Port'Arsa 11, 82100 Benevento, Italy; ^2^Dipartimento delle Scienze Biologiche, Sezione Fisiologia ed Igiene, Università degli Studi di Napoli “Federico II”, Via Mezzocannone 8, 80134 Napoli, Italy; ^3^Dipartimento di Scienze della Vita, Seconda Università degli Studi di Napoli, Via Vivaldi 43, 81100 Caserta, Italy

## Abstract

Peroxisome proliferator-activated receptors (PPARs), which are known to regulate lipid homeostasis, are tightly controlled by nutrient availability, and they control nutrient handling. In this paper, we focus on how nutrients control the expression and action of PPARs and how cellular signaling events regulate the action of PPARs in metabolically active tissues (e.g., liver, skeletal muscle, heart, and white adipose tissue). We address the structure and function of the PPARs, and their interaction with other nuclear receptors, including PPAR cross-talk. We further discuss the roles played by different kinase pathways, including the extracellular signal-regulated kinases/mitogen-activated protein kinase (ERK MAPK), AMP-activated protein kinase (AMPK), Akt/protein kinase B (Akt/PKB), and the NAD+-regulated protein deacetylase SIRT1, serving to control the activity of the PPARs themselves as well as that of a key nutrient-related PPAR coactivator, PPAR*γ* coactivator-1*α* (PGC-1*α*). We also highlight how currently applied nutrigenomic strategies will increase our understanding on how nutrients regulate metabolic homeostasis through PPAR signaling.

## 1. Introduction

### 1.1. PPARs: Nuclear Receptors Functioning as Metabolic Sensors

Energy homeostasis is mostly achieved by hormonal and nutrient-mediated control of the expression of genes encoding metabolic enzymes. Nuclear receptors are responsible for the transcriptional regulation of the vast majority of the aforementioned genes. These receptors are transcription factors that respond to small lipophilic hormones, vitamins, and metabolites. Among the nuclear receptors, the glucocorticoid, thyroid hormone, and estrogen receptors (GR, TR, and ER, resp.) are important regulators of genes involved in metabolic fuel homeostasis both during development and in response to metabolic stress, as well as in the regulation of cellular energy metabolism. Estrogen-related receptors (ERRs) also play critical roles in the regulation of cellular energy metabolism. Other nuclear receptors include the peroxisome proliferator-activated receptors (PPARs), liver X receptors (LXRs), the farnesoid X receptor (FXR), retinoid X receptor (RXR), and hepatocyte nuclear factor-4*α* (HNF-4*α*), all of which are activated by molecules of metabolic pathways, such as lipids and fatty acids (FAs), and thereby function as metabolic sensors. The PPARs take part in the genetic regulation of the complex pathways involved in mammalian metabolism, including fatty acid oxidation and lipogenesis that occur in response to nutritional and physiological stimuli. Taken together, PPAR*α* or NR1C, PPAR*δ* (also known as PPAR*β*) or NR1C2, and PPAR*γ* or NR1C3 constitute group C in subfamily 1 of the superfamily of nuclear receptors [1]. 

### 1.2. PPARs: Structural Features and Interaction with Cofactors

**Figure 1:**
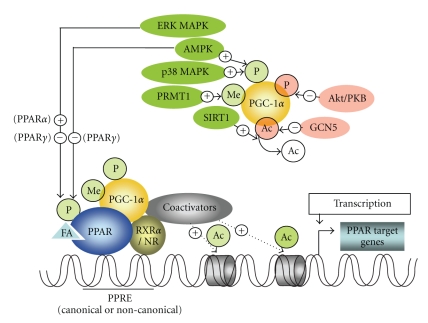
Modulation of the actions of PPAR through phosphorylation by ERK MAPK or AMPK or through regulation of PGC-1*α* activity by various signaling events. Phosphorylation of the PPAR receptors can either increase or decrease their activity. SIRT1-mediated deacetylation activates PGC-1*α*, while acetylation by GCN5 inhibits PGC-1*α*-directed gene expression. Phosphorylation by AMPK or p38 MAPK increases the stabilization of PGC-1*α*, whereas Akt/PKB-mediated phosphorylation facilitates its degradation. PRMT1 activates PGC-1*α* through methylation at several arginine residues. Activation of PGC-1*α* that is recruited to ligand-bound PPAR, the latter being complexed with RXR and/or other nuclear receptors, allows the recruitment of coactivators that acetylate the chromatin, allowing the DNA encoding a particular PPAR target gene to be transcribed. Ac, acetyl group; ERK MAPK, extracellular signal-regulated kinases/mitogen-activated protein kinase; AMPK, AMP-dependent protein kinase; Akt/PKB, Akt/protein kinase B; p38 MAPK, p38 mitogen-activated protein kinase; FA, fatty acid or metabolite from nutrients binding to and activating PPAR; Me, methyl group; P, phosphate group; PGC-1*α*, peroxisome proliferator-activated receptor *γ* coactivator-1*α*; PPAR, peroxisome proliferator-activated receptor; PPRE, PPAR response element; PRMT1, protein arginine methyltransferase 1; RXR, retinoid X receptor; NR, nuclear receptor; SIRT1, sirtuin 2 ortholog 1; +, activation; −, inhibition.

Although the PPARs share high degrees of functional and structural similarities, they are encoded by distinct single-copy genes located on different chromosomes. Human PPAR*α* is located on chromosome 22 [2, 3], PPAR*δ* on chromosome 6 [4], and PPAR*γ* on chromosome 3 [5]. The canonical PPAR response element (PPRE) is a DR1 motif (a direct repeat of the sequence separated by a single nucleotide, preferentially, and adenine [6]) formed by a 5′- and 3′-AGGTCA half-site [7]. This motif typically is present in the promoters of PPAR target genes, including those involved not only in nutrient handling but also in inflammation, cell growth, and differentiation [8, 9]. The formation of complexes between PPARs and other factors is required for the full transcriptional induction of PPAR targets in a variety of tissues. PPARs are predominantly located within the nucleus, where they generally heterodimerize with RXR. In order to achieve their nuclear activity, the PPARs, like all nuclear receptors, have distinct functional domains: an N-terminal domain involved in transcriptional activation, a DNA-binding domain consisting of two zinc-finger motifs, the second finger being involved in binding to the 5′-DR1 half-site as well as dimerization with RXR [6], a hinge region which allows for adequate rotation of the C-terminal domain for interaction with other proteins, and a C-terminal ligand-binding domain including the ligand-dependent activation function- (AF-2), important for RXR heterodimerization and interaction with cofactors [10, 11]. Again, like all nuclear receptors, PPARs act not alone but in association with cofactors that remodel the structure of chromatin in order to either permit or prevent transcription. In the absence of ligand (fatty acids or their derivatives), PPARs form complexes with corepressors such as NCoR, RIP140, or SMRT, which repress transcription through the recruitment of histone deacetylases [12–14]. In the presence of ligand, on the other hand, coactivators, such as p300, CBP, or SRC-1 (all being coactivators related to PPAR function but not primarily associated to nutrient regulation, indicated as coactivators in Figure 1), become bound to the amino terminal of PPAR*γ* coactivator-1 (PGC-1, a key nutrient-modulated coactivator of the PPARs, discussed in Section 3), then acetylate and remodel chromatin, thus enhancing gene transcription via the relief of chromatin condensation (see Figure 1) [15–17]. Depending on the cell type, the ligand-induced conformational changes, and the sequence of the DNA-binding element, a specific complex is formed between the receptor and the coactivators or corepressors, thus allowing fine-tuning of the physiological response. This also explains the variety of changes in gene expression that occur when a nuclear receptor is activated by different ligands. 

### 1.3. Interaction between PPARs and Other Nuclear Receptors

PPARs can associate with other nuclear receptors, and these interactions can involve noncanonical PPREs. Although PPARs and TRs generally compete for interaction with RXR and thus inhibit their respective activity [18], synergism between PPAR and TR can also occur; an example of this is given in the context of nutrient handling, involving the regulation of the expression of the gene encoding uncoupling protein 3, playing a role in the handling of fatty acids within the mitochondria [19]. This interplay has been shown in rat skeletal muscle [20] as well as in cotransfection experiments in rat L6 myoblasts containing a reporter construct driven by the rat UCP3 promoter [20]. Activation of UCP3 gene transcription in vivo by thyroid hormone (T3) requires the presence of fatty acids (the natural ligands of PPARs; see Section 3), while in the absence of fatty acids, transcription can be restored by the PPAR*δ* agonist L165041 [20]. The UCP3 gene promoter has been shown to contain a noncanonical thyroid hormone response element (TRE) termed TRE1 that is conserved from rodents to humans [20, 21], and this response element is also recognized by PPARs [21]. 

### 1.4. PPARs: Different Genes, Different Roles?

It was previously assumed that the three known isoforms, PPAR*α*,*δ*, and *γ*—which display tissue-specific expression (see Table 1) and possess different gene-regulatory profiles—had clearly distinct roles. For instance, PPAR*γ*, expressed predominantly in adipose tissue and the immune system, existing as two distinct proteins *γ*1 and *γ*2, which arise by differential transcription start sites and alternative splicing [5], was assigned the key role as regulator of adipose development, lipid mobilization, and adipose insulin sensitivity [22], whereas PPAR*α*-regulated genes were considered to be associated with lipid oxidation in muscle and liver [23]. The PPAR*α* target genes include carnitine palmitoyltransferase I (CPT I), which is involved in the transport of long-chain fatty acyl groups into the mitochondria, medium-chain acyl-CoA dehydrogenase (involved in *β*-oxidation), and (specifically in liver) mitochondrial 3-hydroxy-3-methylglutaryl-CoA synthase (the rate-limiting enzyme of ketogenesis), as well as peroxisomal acyl-CoA oxidase (peroxisomal *β*-oxidation) and microsomal cytochrome P450 (CYP) FA hydroxylases. Thus, PPAR*α* would be expected to play a critical role in the maintenance of lipid homeostasis (oxidation and production). Although it seems clear that it is primarily involved in lipid metabolism, PPAR*α* may also provide a link between dyslipidemia and diabetes. Exposure of insulin-sensitive tissues (in particular liver and skeletal muscle) to excess nonesterified FA and circulating triglycerides (triacylglycerol, TAG) induces insulin resistance [24], and this can be corrected by the administration of PPAR*α* activators, the actions of which promote the removal of intracellular lipid through FA oxidation [25]. The PPAR*δ* isoform is predominantly expressed in skeletal muscle (where it induces fatty acid oxidation and the expression of largely the same genes as does PPAR*α*), but it is also expressed in brain, heart, liver, adipose tissue, and small intestine [26]. This receptor subtype, which is still under active study, is perhaps the most versatile of the three subtypes, to judge from its wide tissue distribution. PPAR*δ* has been allocated a central role in the direction of fuel usage between different organs (for review, see [27]). 

### 1.5. PPAR Cross-Talk and Fine-Tuning of Nutrient Handling

Because of the overlap in expression profiles between the PPARs [26, 28] (see Table 1), it is perhaps not surprising that there exists cross-talk among PPARs. Indeed, it has been shown that nonliganded PPAR*δ* represses the transcriptional activity of PPAR*α* and PPAR*γ* [29, 30]. Contrasting evidence exists on a nongenomic action of PPAR*δ* on regulation of PPAR*γ* signaling. Using transient transfection studies, it has been shown that the ligand-binding domain of PPAR*δ*, without binding to DNA, exerts ligand-dependent dominant-negative activity on PPAR*γ*1 signaling [30], although in a previous study a non-DNA-binding PPAR*δ* derivative failed to exert such an effect [29]. Since the PPARs act as RXR heterodimers, it is conceivable that, in analogy to ligand-dependent RXR competition between PPAR and liver X receptor (LXR) [31, 32], PPAR*δ* and LXR*α*  [33], PPAR*α* and TR [34], as well as PPAR*γ* and TR [35], competition for RXR could occur between the PPAR isoforms. Indeed, the nongenomic dominant-negative effect of PPAR*δ* on PPAR*γ* is likely to involve RXR sequestration, thus preventing PPAR*γ*-RXR heterodimerization [30]. Interaction between PPARs and other nuclear receptors and PPAR cross-talk together allow for fine-tuning of interorgan nutrient handling, in concert with the effects of the various signaling molecules that are common to the nuclear receptors and that direct their actions.

## 2. Nutritional Control of Expression and Activation of PPARs

### 2.1. Nutrients: Natural PPAR Ligands

PPARs have been shown to be under nutritional control. Dietary nutrients and their derivatives (or adipose-derived fatty acids during food deprivation) directly control PPAR activity since they are the natural ligands of PPARs. PPARs display the greatest preference for monounsaturated and polyunsaturated fatty acids (MUFAs and PUFAs, resp.), as demonstrated by means of various ligand-binding assays [36, 37]. The fact that each PPAR activates a different gene program, despite their overlapping expressions, would seem to suggest that PPARs display ligand specificity. Indeed, the structure of the ligand-binding pocket differs considerably among the various PPARs, as revealed by X-ray crystal-structure analysis [37, 38]. Despite this, natural fatty acids can be ligands of all three PPAR isoforms. Using comparative nutrigenomics analysis, it has been recently shown that, in response to high-fat diet, the diet-induced target genes of PPAR*α* are conserved between yeast, mouse, rat, and man, underlining the importance of nutritional control of PPAR function [39]. A different nutrigenomic approach consisted of the use of synthetic triglycerides composed of one single fatty acid in combination with gene expression profiling to examine the effects of various individual dietary fatty acids on hepatic gene expression in mice. Results revealed that (i) increased fatty acid chain length and degree of unsaturation increased the number of genes being upregulated and that (ii) genes regulated by dietary unsaturated fatty acids remained unaltered in PPAR*α* knockout mice, identifying PPAR*α* as their target, and the same genes were upregulated in mice treated with the PPAR*α* agonist WY14643 [40]. Since the binding of a ligand promotes a conformational change that is permissive for interactions with tissue-specific coactivator proteins (see Section 3), allowing nucleosome remodeling and activation of the transcription of cell type-specific target genes [37, 41], it is conceivable that upon binding to a ligand-binding pocket a given fatty acid induces conformational changes, which differ among the various PPAR subtypes. Given that the transcriptional activity induced by each PPAR subtype is cell type specific [42], the different conformations induced following ligand binding might confer cell specificity on the various PPARs (through heterodimerization with different receptors and binding to cell type-specific cofactors). 

### 2.2. Role of PPARs in the Adaptation to Nutrient Deprivation

One widely employed way of studying how PPAR expression and function is controlled by nutrients in different tissues is by imposing nutrient deprivation. The fasting state influences the actions of all known PPARs. For instance, fasting is known to increase PPAR*α* signaling in the liver, through increased mRNA levels of the coactivator PGC-1*α* [43], and thereby tightly to regulate hepatic gluconeogenesis and FA oxidation. Interestingly, and perhaps paradoxically, it has recently been shown that during fasting upregulation of gene expression by PPAR*δ*, not PPAR*α*, is sensitive to adipose-derived plasma FA, thus assigning a clear role for PPAR*δ* as a plasma FA sensor in liver [44]. Given the central role of PPAR*δ* in controlling skeletal muscle lipid utilization, fasting (which results in a greater reliance on fatty acids) would be expected to increase PPAR*δ* expression and/or activity. In line with this, starvation has been shown to result in a dramatic but transient upregulation of PPAR*δ* mRNA in rat gastrocnemius muscle [45]. This correlated with rapid nuclear accumulations of PPAR*δ* and the coactivator PGC-1*α* after food deprivation [45]. It has also been shown that, in skeletal muscle, PPAR*δ* and PGC-1*α* physically interact with each other within the nucleus [46]. This interaction would then lead both to increased fatty acid levels and to increased expression of genes such as those for myosin heavy chain I (MHC I), thioesterase I (MTE I) [47], and carnitine palmitoyl transferase 1 (CPT1), as well as uncoupling protein 3 (UCP3) [48, 49], thereby underlining the role of PPAR*δ* as a key regulator of muscle-fiber switching and fatty acid metabolism. The transient nature of the increased PPAR expression that occurs during starvation (both the mRNA levels and the nuclear accumulation decreasing once serum FA levels increase [45]) would imply that excessive intracellular fat accumulation inhibits PPAR expression. Indeed, rat PPAR*α* and PPAR*δ* mRNA are each shown to be downregulated after a 48-hour fast [45] and conversely suppression of free fatty acids (using the antilipolytic drug nicotinic acid) was found to increase the mRNA levels of skeletal muscle PPAR*δ* [50]. Both the early fasting state and nicotinic acid treatment cause increased phosphorylation of AMP-activated protein kinase [45], which is known to interact functionally with, and stimulate, PPAR (see Section 3). Once serum fatty acid levels become elevated during food deprivation, the raised muscle AMPK phosphorylation level falls. Taken together, these data indicate that the initial absence of burnable fatty acids during food deprivation triggers a process in which the myocyte is rapidly “converted” to a cell type dedicated to the uptake and burning of fat. Once the intracellular FA levels reach a certain value, the myocyte switches off AMPK and reduces PPAR expression throughout the remainder of the starvation period. Similarly, a downregulation of PPAR*δ* mRNA levels in human skeletal muscle expression has been reported in healthy human subjects after a 48-hour fast [51]. Although data from shorter food-deprivation periods in humans are lacking, it seems likely that, after food deprivation, there is a rapid but transient increase in skeletal muscle PPAR expression in humans too. Activation of the white adipose tissue PPAR*γ* promotes lipid synthesis and storage [52]. Therefore, during fasting, the action of PPAR*γ* has to be inhibited. Indeed, in mature adipocytes, during fasting, its action is inhibited by a physical interaction with the protein sirtuin 1 (SIRT1) (the mammalian Sir2 ortholog), the result being lipolysis [53]. In contrast, the action of PPAR*γ* is enhanced by direct binding to lipin-1, a protein which is known to promote triacylglycerol storage within adipocytes, and to be less expressed during fasting, resulting in lipolysis [54]. There exists cross-talk between PPAR*γ* and PPAR*δ* in this context: PPAR*δ* inhibits the control of PPAR*γ* expression [55], enhancing lipolysis.

### 2.3. Influence of the Nutritional State on PPAR Action

**Figure 2:**
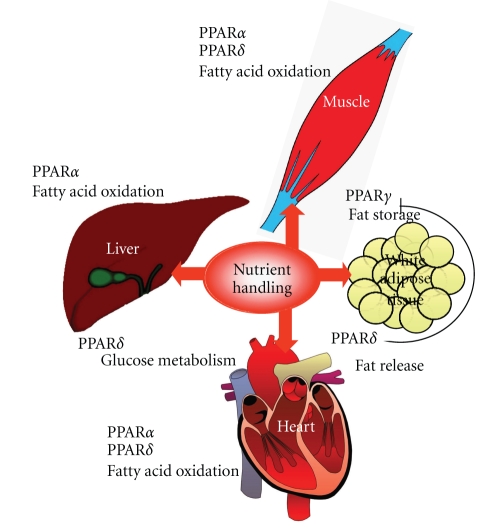
PPAR action in relation to nutrient handling in metabolically active tissues.

It is well known that in the liver PPAR*α* controls fatty acid oxidation [23], but the role of PPAR*δ* in the liver has up to now scarcely been assessed. A recent transcriptional profiling analysis using PPAR*α*- versus PPAR*δ*-depleted mice has revealed that PPAR*δ* exerts a distinct role in the liver, namely in the control of glucose utilization and lipoprotein metabolism, as well as the suppression of inflammation [56], whereas PPAR*α* mainly controls hepatic lipid homeostasis, which was especially revealed during fasting, causing drastic changes in the hepatic gene expression profile of the PPAR*α*-depleted mice but not in that of the PPAR*δ*-depleted mice [56]. It should, however, be noted that, as described above, the same group assigned a predominant role for PPAR*δ* as FA sensor in liver during fasting, being activated in response to a rise in plasma FA levels, in contrast to PPAR*α* [44]. It is conceivable that dietary FAs and adipose-derived FAs may activate different PPAR-related pathways, and more research is clearly necessary to gain more insight into the respective roles of PPAR*α* and PPAR*δ* in nutrient handling in liver and other organs. In Figure 2, an overview of PPAR action on nutrient handling in the different metabolically active organs, on the basis of the above data, is depicted. 

## 3. Nutrient Availability-Related Cytosolic Signaling Pathways Directly and Indirectly Modulating PPAR Activity

### 3.1. Control of PPAR Phosphorylation by Upstream Signaling Related to Fuel Use

Activation of cytosolic and nuclear signaling, modulating the activity of PPARs, is nutrient regulated. Recent data have revealed that kinases whose activity is modulated by the nutritional state can directly act on phosphorylation of PPARs. Insulin increases phosphorylation and transcriptional activity of PPAR*α*, due to increased activity of the extracellular signal-regulated kinases/mitogen-activated protein kinase (ERK MAPK) pathway [57]. This same pathway inhibits adipogenesis through phosphorylation and inactivation of PPAR*γ* [58]. Both actions are in favor of an increase in insulin sensitivity. Adenosine monophosphate-activated protein kinase (AMPK) is an energy sensor that is activated when the cell*-*energy level is low [59]. Once activated, AMPK stimulates both glucose uptake and lipid oxidation to produce energy, while turning off energy-consuming processes. Direct proof for phosphorylation of PPAR*α* by AMPK has not been provided thus far, but it has been shown that phosphorylation of PPAR*γ* by AMPK represses both the ligand-dependent and -independent transcriptional activation function of the receptor [60], thus counteracting adipogenesis and favoring lipid oxidation. PPAR*δ* phosphorylation and activity are also likely to be directly controlled by kinase pathways, but to our knowledge evidence for this has not been obtained yet. 

### 3.2. Nutrient-Related Control of PPAR Activity through Activation of PGC-1

The aforementioned kinases as well as other kinases and proteins, functionally associated with nutrient availability, do not only act directly on the PPARs but also modulate the activity of important regulators of the activity of PPARs, namely the PGC-1s, well known as key transcriptional coactivators involved in the control of nutrient and energy metabolism. Three isoforms of PGC-1 are known [namely, PGC-1*α*, PGC-1*β*, and PGC-1-related coactivator (PRC)]. They each control mitochondrial physiology and FA oxidation, while exerting an isoform-specific regulation of different metabolic pathways [61]. PGC-1*α* and PGC-1*β* are highly expressed in heart, skeletal muscle, and brown adipose tissue, where, via nuclear respiratory factors (NRFs), they induce expression of genes involved in the regulation of mitochondrial biogenesis [61]. PGC-1 family members can interact not only with PPARs but also with other members of the nuclear receptor superfamily, such as ERR, LXR, and HNF-4*α* [62–64], and with distinct transcription factors and regulatory elements, including cAMP response element-binding protein (CREB), the lipogenic transcription factor sterol regulatory element-binding protein-1c (SREBP-1c), and forkhead box O1 (FOXO1) [43, 65–68]. PPARs, ERR, HNF-4*α*, and GR commonly bind to LXXLL motifs present in the N-terminal domain of PGC-1, while other transcription factors bind to different regions of the protein, hence allowing a coordinated transcriptional response to nutrient and physiological signals. Although at least two clinical studies have identified a correlation between mutations of the gene encoding PGC-1*α* and either insulin resistance or diabetes [69, 70], basic research has produced contrasting results. For instance, overexpression of PGC-*1* *α*, leading to increased PPAR*α* expression in primary cultures of rat skeletal muscle cells, induces increased expression of the mammalian tribbles homolog TRB3, an inhibitor of Akt signaling [71], a result that implies that PGC-1*α* has the potential to cause insulin resistance through PPAR*α* signaling. Moreover, PGC-1^−/−^ mice are protected against the insulin resistance induced by a high-fat diet [72].

## 4. Nutrient-Related Factors Controlling PGC-1 Activity

In response to nutrient signaling, (e.g., in the fasting/fed state, discussed in Section 2), PGC-1 isoforms regulate their own transcription, but a posttranslational regulation also occurs. In particular, (i) phosphorylation, (ii) reversible acetylation, and (iii) methylation are key mechanisms by which the function of PGC-1*α* function is maintained. (i) Three protein kinases directly phosphorylate PGC-1*α*. P38 mitogen-activated kinase (P38 MAPK) phosphorylates PGC-1*α* [73], leading to a more active and stable protein that is unable to bind the p160 corepressor. Also, AMPK phosphorylates and activates PGC-1 [59] (Figure 1). Activation of PGC-1*α* by phosphorylation through p38 MAPK and AMPK occurs in muscle [74, 75] whereas, in contrast, phosphorylation of PGC-1*α* in the liver by Akt/protein kinase B (Akt/PKB, which is a central kinase in the insulin-signaling cascade) leads to decreased stability and activity (see [76]; Figure 1). It is noteworthy that AMPK is constitutively activated in the muscles of transgenic mice harboring an activated form of PPAR*δ* [77], while skeletal muscle cells exposed to a pharmacological PPAR*δ* activator show increased AMPK activity [78, 79]. (ii) By analogy with the antagonistic phosphorylation induced by AMPK and Akt/PKB, the protein deacetylase SIRT1 opposes the action of GCN5, an acetyl transferase. GCN5 inhibits PGC-1 transcriptional activity by acetylating PGC-1 at several lysine residues [80, 81] while SIRT1 activates PGC-1 by deacetylating it and induces expression of PGC-1 gene targets (see [82, 83]; Figure 1). SIRT1, located within the cell nucleus, modulates gene expression in ways that depend on the cellular energy state, which it senses through the cell's NAD+ levels. SIRT1 is an important regulator of those metabolic processes that are initiated by a rise in the intracellular NAD+/NADH ratio when the energy supply is low. Recently, SIRT1-mediated deacetylation of PGC-1*α* was reported to play a critical role in the regulation of hepatic FA oxidation: nutrient signaling involving SIRT1 and PGC-1*α* activated gluconeogenic and fatty acid oxidation genes in the fasting liver [82]. Such nutrient signaling gives rise to increases in pyruvate and NAD+ levels, resulting in increases in the amount and enzymatic activity of SIRT1. Loss of SIRT1 from hepatocytes impairs PPAR*α* signaling, resulting in decreased fatty acid oxidation and leading to the development of hepatic steatosis on a high-fat diet, whereas overexpression of SIRT1 induces expression of PPAR*α* gene targets [84]. SIRT1 induces PPAR*α* signaling through deacetylation of PGC-1*α* without affecting the formation of the PPAR*α*-PGC-1*α* complex: in SIRT-knockdown hepatocytes, PGC-1*α* is still recruited to the PPAR response element (PPRE) of FA oxidation genes [84], but it remains acetylated and thus unable to induce transcription of PPAR*α* gene targets. SIRT1 has been identified as a functional regulator of PGC-1*α* that induces a metabolic gene transcription program of muscle mitochondrial function and fatty acid oxidation during fasting [82]. Thus, consistent with a switch from glucose to fatty acid oxidation that occurs in nutrient-deprivation states, SIRT1 is required for induction and maintenance of fatty acid oxidation in response to low glucose concentrations. Importantly, the action of SIRT1 on PGC-1 differs among stimuli, deacetylating PGC-1*α* only in response to nutrient signaling, not to glucagon [82, 85]. In addition, whereas SIRT1 overexpression protects against both the hepatic steatosis and glucose intolerance induced by high-fat feeding [86, 87], oligonucleotide knockdown of hepatic SIRT1 in a rat model of T2DM has been reported to reduce hyperglycemia by normalizing basal hepatic glucose production and increasing hepatic insulin sensitivity, leading to the suggestion that novel SIRT1 inhibitors targeted at the liver might prove beneficial in the treatment of T2DM [88]. (iii) Protein arginine methyltransferase I (PRMT1; Figure 1) coactivates nuclear receptors [89] and has been reported to induce the PGC-*1* *α* function through methylation at several arginine residues in the C-terminal region [89]. Further, it has recently been demonstrated that impaired PRMT1 activity may be implicated in glucose intolerance in nonobese diabetic Goto-Kakizaki rats through disturbed hepatic glucose metabolism and insulin secretion [90]. An overview of the above discussed nutrition-related kinases and enzymes modulating PPAR activity directly or via PGC-1 is given in Figure 1. 

## 5. Conclusions

The beginning of this century saw a rapid expansion of research on the obesity-counteracting potential of PPARs. It is important to consider that dietary changes can drastically interfere with interorgan signaling, and that PPARs can play an important role in this context. Excess fat supply and nutrient deprivation, followed by analysis utilizing comparative nutrigenomics and transcriptional profiling, have rapidly increased our knowledge on nutrient-mediated regulation of PPAR action, and has revealed that nutrient-based regulation of PPAR action is conserved from yeast to man. PPAR action is under tight control of upstream signaling through factors such as SIRT1 and AMPK, having PGC-1 as a target, and this signaling is also influenced by the nutritional state. The further application of potent transcriptomic profiling techniques under different dietary conditions will help to establish which natural ligands might activate each PPAR in a given cellular context. This research will be important since it will allow us to assess how even subtle changes in our daily diet can cause drastic variations in PPAR-mediated metabolic homeostasis. Normalization of lipid and glucose metabolism may be achieved via pharmacological modulation of PPAR activity but the obtained results are not always beneficial (not discussed in this review), which indicates that our knowledge on the intricate network controlling PPAR action is still far from complete. The challenge for future research is to unravel the complex nutrient-influenced metabolic signaling involving the aforementioned factors, in order to be able to “safely” interfere with these processes, as an important step to relieve the burden of obesity and its related disorders. 

## Figures and Tables

**Table 1:** Tissue distributions of the various PPARs (RNA and protein) in adult rodents and humans. Abbreviations: GI, gastrointestinal; WAT, white adipose tissue; BAT, brown adipose tissue; CNS, central nervous system. Symbols: −, absent; ±, barely detectable; +, weak; ++, moderate; +++, high; ++++, very high (Taken from [26, 28, 45]).

Tissue	Protein/mRNA	PPAR*α*	PPAR*β*/*δ*	PPAR*γ*
*GI tract*				
Mouse	Protein		++++	
Human	mRNA	+ or ++	+ to ++++	++ to ++++
Mouse	mRNA	+ or +++		
Rat	mRNA	++ to ++++	++ to ++++	+ to ++

*Liver*				
Mouse	Protein		+++	
Rat	Protein	++++		
Human	mRNA	+ to ++++	+ or ++	+ to ++
Mouse	mRNA	++++		
Rat	mRNA	++++	++	−

*Kidney*				
Mouse	Protein		+++	
Human	mRNA	++ to ++++	+ to +++	+ to +++
Mouse	mRNA	+++		
Rat	mRNA	+++ to ++++	+++	±

*Heart*				
Mouse	Protein		++	
Human	mRNA	+++ to ++++	+ or +++	++ to +++
Mouse	mRNA	++		
Rat	mRNA	+ or +++	+	±

*WAT*				
Human	mRNA	+	++	++++
Rat	mRNA	+	++	+++

*BAT*				
Mouse	mRNA	+++		
Rat	mRNA	++++	++	++++

*CNS*				
Rat	mRNA	+	+ or +++	±

*Brain*				
Mouse	Protein		++	
Human	mRNA	++	+ or +++	
Rat	mRNA	+	+++	

*Skeletal muscle*				
Mouse	Protein		+	
Human	mRNA	++ to ++++	++ or ++++	+ or +++
Mouse	mRNA	+++	+++	±
Rat	mRNA	++	++++	±

*Skin*				
Mouse	Protein		+++	
Rat	mRNA	+	±	

*Lung*				
Mouse	Protein		++	
Human	mRNA	+	+ or +++	++++
Rat	mRNA	+	++	+
